# Electroacupuncture at ST36 Increases Bone Marrow-Derived Interstitial Cells of Cajal via the SDF-1/CXCR4 and mSCF/Kit-ETV1 Pathways in the Stomach of Diabetic Mice

**DOI:** 10.1155/2018/7878053

**Published:** 2018-01-23

**Authors:** Jiao Zhao, Jing An, Shi Liu

**Affiliations:** Division of Gastroenterology, Union Hospital, Tongji Medical College, Huazhong University of Science and Technology, Wuhan, China

## Abstract

**Background:**

The loss of interstitial cells of Cajal (ICC) is observed in diabetic gastroparesis. Electroacupuncture (EA) maintains ICC networks, but the effects and mechanisms of EA on ICC of bone marrow derivation in the stomach have not been investigated.

**Methods:**

C57BL/6 mice were randomized into six groups: control, diabetic (DM), bone marrow transplantation (BMT) + DM, BMT + DM + sham EA (SEA), BMT + DM + low-frequency EA (LEA), and BMT + DM + high-frequency (HEA). c-Kit^+^GFP^+^ cells in the stomach were detected by immunofluorescence staining. Western blotting and qRT-PCR were employed to determine c-Kit, GFP, SDF-1, CXCR4, mSCF, pERK, and ETV1 expression.

**Results:**

(1) c-Kit^+^GFP^+^ cells were elevated in the BMT + DM + LEA and HEA groups. (2) The mRNA and protein levels of GFP, SDF-1, and CXCR4 were increased in the BMT + DM + LEA and BMT + DM + HEA groups. (3) The mRNA and protein levels of mSCF, c-Kit, pERK, and ETV1 were significantly reduced in the DM group but markedly elevated in the BMT + DM + LEA and HEA groups.

**Conclusion:**

EA at ST36 increases bone marrow-derived ICC in the stomach of diabetic mice via the SDF-1/CXCR4 and mSCF/c-Kit-ETV1 pathways.

## 1. Introduction

Gastroparesis, characterized by delayed gastric emptying in the absence of mechanical obstruction, is one of the most common complications of diabetes mellitus, which seriously affects the normal work and life of patients. Although various treatment interventions with or without pharmaceutical compounds have been tested, treatment of gastroparesis remains unsatisfactory. Electroacupuncture (EA), an advanced form of traditional acupuncture, has promising therapeutic effects. Several studies have demonstrated that EA at ST36 promotes gastric emptying and improves symptoms in gastroparesis patients and animal models [1, 2]. However, the detailed mechanism underlying these effects is not yet fully understood.

Loss or dysfunction of ICC is associated with gastric motor disorders. Reduced ICC networks have been identified in gastroparesis in both humans and animal models [3]. However, it is unknown whether EA has an impact on ICC. Our previous findings and those from other studies have shown that EA maintains ICC networks [4, 5]. Apoptosis leads to the loss of ICC; proliferation and replenishment from stem cells restore and increase ICC numbers. In our previous study, EA at ST36 sustained ICC networks by inhibiting cellular apoptosis and enhancing cellular proliferation in the stomach of diabetic rats [4]. However, little is known about the effects of EA on ICC derived from stem cells.

Bone marrow-derived cells (BMDCs) differentiate into various types of cells such as neurons, hepatocytes, cardiomyocytes, and epithelial cells [6]. In recent years, a small number of studies have shown that BMDCs differentiate into ICC of the intestinal tract [7, 8]. Furthermore, studies have shown that EA promotes the differentiation and migration of BMDCs, thereby repairing neurons after spinal cord injury [9]. However, whether EA promotes the differentiation of BMDCs into ICC in the stomach remains to be demonstrated.

BMDCs migrate to sites of injury. The migration of stem cells towards injured tissue is directed by various chemokines [10]. Stromal cell-derived factor 1 (SDF-1) and C-X-C motif chemokine receptor 4 (CXCR4) are essential for mobilization and homing of stem cells [11]. However, it is not yet known whether EA has an effect on the SDF-1/CXCR4 signaling pathway during the migration of BMDCs to the stomach.

ICC expresses the receptor tyrosine kinase c-Kit, an established ICC marker. The stem cell factor (SCF)/Kit signaling pathway plays an important role in the development and maintenance of normal ICC networks [12, 13]. Ets variant 1 (ETV1) is a survival factor expressed by ICC that is required for the maintenance of normal ICC networks, and it is stabilized by physiological levels of c-Kit [14]. According to our previous study, EA maintained ICC via membrane-bound SCF (mSCF)/Kit-ETV1 signaling in diabetic mice [15]. However, the effect of EA on mSCF/Kit-ETV1 signaling in chimeric diabetic mice is unknown.

The aims of our study were to (1) investigate whether EA affects bone marrow-derived ICC in the stomach, (2) assess the effects of EA on the SDF-1/CXCR4 signaling pathway during the migration of BMDCs to the stomach, and (3) evaluate the effects of EA on the mSCF/Kit-ETV1 signaling pathway in chimeric diabetic mice.

## 2. Materials and Methods

### 2.1. Ethics Statement and Animals

The mice received humane care, and this study was carried out strictly in accordance with the recommendations in the Guide for the Care and Use of Laboratory Animals from the National Institutes of Health. The study protocol was approved by the Institutional Animal Care and Use Committee (IACUC) of Tongji Medical College, Huazhong University of Science and Technology.

Six- to eight-week-old male green fluorescent protein- (GFP-) transgenic C57BL/6 mice were used as donors and were purchased from Model Animal Research Center of Nanjing University (Nanjing, China). This GFP-transgenic mouse line expresses an enhanced GFP cDNA under the control of a chicken beta-actin promoter and cytomegalovirus enhancer in all tissues except erythrocytes and hair [16]. Male wild-type C57BL/6 mice (6 to 8 weeks old) were used as recipients and were purchased from Beijing HFK Biotechnology Co., Ltd. (Beijing, China). All animals were housed under normal laboratory conditions (22°C and 12/12-h light/dark cycle) and kept in a specific pathogen-free (SPF) environment. Food and water were available ad libitum. The mice were acclimated to the laboratory environment for one week before the experiment began.

### 2.2. Experimental Protocols

#### 2.2.1. Randomization

Sixty-six C57BL/6 mice were randomly divided into six treatment groups: control, diabetic (DM), bone marrow transplantation (BMT) plus diabetic (BMT + DM), BMT plus diabetic plus sham EA (BMT + DM + SEA), BMT plus diabetic plus low-frequency EA (BMT + DM + LEA), and BMT plus diabetic plus high-frequency EA (BMT + DM + HEA).

#### 2.2.2. Bone Marrow Transplantation

GFP-transgenic C57BL/6 mice were anesthetized with pentobarbital sodium (50 mg/kg) and euthanized by cervical dislocation for bone marrow isolation. Bone marrow cells were harvested under aseptic conditions by flushing the cavity of the femur and tibia of GFP-transgenic mice with Dulbecco's modified Eagle's medium (DMEM). The bone marrow samples were filtered with 45-*μ*m nylon mesh to remove debris. The red cells were lysed using red blood cell lysis buffer (Biosharp, Hefei, China). Cell concentrations were determined. The cells were resuspended in DMEM at a concentration of 1 × 10^7^ cells/350*μ*l, which was the transplantation volume for each recipient. Wild-type C57BL/6 mice were given drinking water with antibiotics (280 mg erythromycin and 320 mg gentamicin sulfate per liter of drinking water) for 10 days before irradiation and 2 weeks after irradiation. The mice were exposed to 9 Gy of total-body irradiation. Then, bone marrow cells were injected into irradiated C57BL/6 mice via the tail vein. Mice in the control and DM groups were injected with the same volume of DMEM via the tail vein. After transplantation, the weight, stool, hair, and mental state of mice were assessed daily for two weeks. The extent of chimerism was detected by flow cytometry after four weeks. The mice were used for subsequent experiments only when 80% or more of their blood cells had been replaced with GFP^+^ cells.

#### 2.2.3. Induction of Diabetes

Four weeks after transplantation, diabetes was induced by a single intraperitoneal injection of streptozotocin (STZ, 150 mg/kg; Sigma, St. Louis, MO, USA), which was freshly dissolved in 0.1 mol/l citrate buffer solution (pH = 4.5; Sigma). The mice were fasted overnight prior to STZ injection. Mice in the control group were injected with the same volume of citrate buffer. One week later, mice were regarded as diabetic if their blood glucose levels were >16.7 mmol/l.

#### 2.2.4. EA Stimulation

The acupoint ST36 is located approximately 2 mm below the fibular head at the posterolateral knee of bilateral hind limbs [17]. For the real EA group, needles (7 mm in length and 0.16 mm in diameter; Beijing Zhongyan Taihe Medical Instrument Co., Ltd., Beijing, China) were bilaterally inserted to a depth of 2-3 mm at ST36 and connected to the EA device (G6805-2A, Shanghai Huayi Medical Instrument Factory, Shanghai, China). The stimulation parameters for LEA were 10 Hz, 1 mA, and 30 min, while the parameters for HEA were 100 Hz, 1 mA, and 30 min. The electrical current was administered until the bilateral hind limbs started to tremble slightly. For the SEA group, needles were inserted into acupoint ST36 without electrical current for 30 min. EA was performed daily at ST36 for eight weeks. These parameters were selected based on our preliminary experiments, which suggested stimulatory effects on the gastric motor and ICC [4, 18]. To eliminate the influence of stress, animals were restrained in a cage for 30 min/d before EA for two weeks.

#### 2.2.5. Preparation of Specimens

After stimulation for eight weeks, the mice were euthanized to harvest their stomach tissue. A piece of the fresh tissue was used for immunofluorescence staining. The remaining tissues were stored at −80°C for western blotting and real-time quantitative reverse-transcriptase PCR (qRT-PCR) analyses. Blood specimens obtained from mouse hearts were centrifuged, and serum samples were stored at −80°C for enzyme-linked immunosorbent assay (ELISA).

### 2.3. Fluorescence-Activated Cell Sorting

Bone marrow cells were obtained according to the method described for bone marrow transplantation. Approximately 50*μ*l of blood from each chimeric mouse was directly collected from the tail vein using heparin-coated EP tubes. Red blood cells were lysed using red blood cell lysis buffer. Each sample was washed three times with PBS. The cells were resuspended in PBS containing 2% paraformaldehyde (PFA). The cells were then immediately analyzed using a flow cytometer (FACScan; Becton Dickinson, Mountain View, CA, USA). Fluorescence-activated cell sorting (FACS) of each chimeric mouse sample was performed to evaluate the extent of GFP chimerism in the mice.

### 2.4. Gastric Emptying

Gastric emptying was performed by a modification as described previously [19]. Five mice per group were used to measure gastric emptying. Mice were fasted overnight and allowed free access to water. The test meal, containing phenol red (0.5 mg/ml) as an indicator and carboxymethylcellulose (15 mg/ml), was continuously stirred and then held at 37°C. Mice received test meals at a volume of 300*μ*l by gavage. Twenty minutes after gavage, the mice were sacrificed. The whole stomach was removed carefully after ligation at the cardia and pyloric; then the stomach was opened and its contents were poured into a test tube and washed with 4 ml of distilled water. At the end of the experiment, NaOH solution (2 ml, 1 M) was added to each tube to develop the maximum color intensity. The absorbance of the sample read at 560 nm with a spectrophotometer (HITACHI, U-2900) reflected the amount of phenol red remaining in the stomach. The rate of gastric emptying was calculated according to the following formula: Gastric emptying (%)  =  100 × (1 − *X*/*Y*). *X* represents the absorbance of phenol red collected from the stomachs of animals sacrificed 20 min after the test meal. *Y* represents the absorbance of phenol red recovered from the stomachs of control animal sacrificed immediately after the administration of the test meal.

### 2.5. Immunofluorescence Staining

Tissues from six mice per group were used for immunofluorescence staining. Freshly isolated mouse stomach specimens were immersed in ice-cold Krebs solution previously bubbled with 95% O_2_ and 5% CO_2_ (mmol/l): 118.1 NaCl, 4.8 KCl, 25 NaHCO_3_, 1.0 NaH_2_PO_4_, 1.2 MgSO_4_, 11.1 glucose, and 2.5 CaCl_2_ at pH 7.3–7.4. The stomach was opened along the lesser curvature, and the gastric contents were washed away with ice-cold Krebs solution. The tissue was then pinned onto a dish that was coated with Sylgard. The mucosa and submucosa were carefully peeled away with forceps, and only the* tunica muscularis* of the stomach was used. The stomach was fixed with ice-cold 4% PFA for ten minutes. Following fixation, the preparations were rinsed three times (ten minutes each time) in 1x PBS. Nonspecific binding was blocked by treatment with normal goat serum and 0.3% Triton X-100. The tissues were next incubated for 48 h at 4°C with primary rat monoclonal antibodies against c-Kit (1 : 100; #14-1172-81, eBioscience, San Diego, CA, USA) and rabbit polyclonal antibodies against GFP (1 : 1000, #ab6556, Abcam, Cambridge, UK) diluted in primary antibody dilution buffer containing 0.3% Triton X-100. After washing with PBS, immunoreactivity was detected by incubation with Dylight 488 with goat anti-rabbit IgG (1 : 200, #A23220, Abbkine, CA, USA) and Dylight 594 with goat anti-rat IgG (1 : 200, #A23440, Abbkine) diluted in PBS containing 0.5% Triton X-100 for 2 h. A confocal microscope (Olympus, Tokyo, Japan) was used to examine the specimens.

### 2.6. Western Blotting

Fresh-frozen stomach tissues from five mice per group were thawed and homogenized in RIPA buffer (Beyotime, Shanghai, China) with protease inhibitor (Beyotime). The mixtures were centrifuged at 12000*g* for 15 min at 4°C, and the supernatants were collected as total protein. The protein concentrations of the supernatants were measured using the bicinchoninic acid (BCA) method. Standards of different concentrations were prepared by making serial dilutions. A total of 200*μ*l of the BCA Working Reagent was added to 25*μ*l of each BSA protein standard, blank, and unknown sample. The mixtures were vortexed gently to mix thoroughly and then incubated at 37°C for 30 min. The protein assay was performed using a microplate photometer at a wavelength of 570 nm. The protein concentration was calculated according to the standard curve. Equivalents of 120*μ*g of extracted proteins were separated via 10% sodium dodecyl sulfate-polyacrylamide gel electrophoresis (SDS–PAGE) and transferred to polyvinylidene fluoride (PVDF) membranes (Millipore, Bedford, MA, USA). Nonspecific binding sites were blocked with 8% nonfat dry milk in Tris-buffered saline containing 0.1% Tween 20 (TBST) at room temperature for 1 h. Then, the membranes were incubated with primary polyclonal goat anti-c-Kit (1 : 1000; #AF1356, R&D Systems, Minneapolis, MN, USA), goat anti-SCF (1 : 1000; #AF-455-NA, R&D Systems), rabbit anti-ERK (1 : 1000; #9102, Cell Signaling, Danvers, MA, USA), rabbit anti-pERK (1 : 1000; #9101, Cell Signaling), rabbit anti-ETV1 (1 : 500; #ab81086, Abcam), rabbit anti-GFP (1 : 1000; #ab6556, Abcam), rabbit anti-SDF-1 (1 : 500; #GTX116092, GeneTex, Irvine, CA, USA), or rabbit anti-CXCR4 (1 : 500; #11073-2-AP, Proteintech, Rosemont, IL, USA) antibodies overnight at 4°C. Rabbit anti-GAPDH antibodies (1 : 3000; #ANT012s, Antgene, Wuhan, China) served as the internal control. After three washes with TBST, the membranes were incubated with horseradish peroxidase- (HRP-) linked secondary rabbit anti-goat (1 : 5000; #ANT021s, Antgene) and goat anti-rabbit (1 : 5000; #ANT020s, Antgene) antibodies for 1 h at room temperature. After washing three times in TBST, the bands were detected via a chemical reaction with enhanced chemiluminescence reagent (ECL; ThermoFisher, Rockford, MA, USA), and the blot was subjected to autoradiography. Quantity One software (Bio-Rad Technical Service Department, version 4.6.2) was used to measure the band intensities.

### 2.7. ELISA

Approximately 1 ml of blood was obtained from six mice per group. The serum concentration of SDF-1 was determined using an ELISA kit (R&D Systems). ELISA was performed according to the manufacturer's procedure.

### 2.8. Reverse Transcription and Quantitative PCR

Tissues from five mice were used for qRT-PCR. Total RNA was extracted from stomach tissues with an RNA extraction kit (Aidlab, Beijing, China) according to the manufacturer's instructions. Approximately 1*μ*g of total RNA was used for reverse transcription using a First Strand cDNA Synthesis Kit (Takara, Otsu, Japan). qRT-PCR was performed using SYBR Premix Ex TaqII (Takara) on a Roche LightCycler® 480 (Roche, Basel, Switzerland). The following specific primers were used: GFP, CAGAAGAACGGCATCAAGGTG and CGGACTGGGTGCTCAGGTAG; c-Kit, CAGAGGCTTAGCGGAGTGAA and AGGGCAAGGACAAGGGAAC; mSCF, GGAAAATAGTGGATGACCTCGTG and TGGAATCTTTCTCGGGACCTAAT; ERK, TTCAGCAACAGGCTCATC and TGTTCAGGAGGAGGTTTGATGGC; ETV1, TTCATTGCCTGGACTGGACG and TGCCTTGCTTGACGGGTTA; SDF-1, CAGTCAGCCTGAGCTACCGA and GAGGGAGGAGCGAGTTACAAA; CXCR4, AGAAGCTAAGGAGCATGACGG and GCGTGGACAATAGCGAGGT; and GAPDH, CATCACTGCCACCCAGAAGA and TGAAGTCGCAGGAGACAACC. GAPDH was used to normalize the amount of cDNA in each sample. Assays were repeated at least three times. The 2^−ΔΔCT^ method was used to quantify relative changes in gene expression.

### 2.9. Statistical Analysis

The mean value was used for statistical analyses. All data are shown as the mean ± SE, and ANOVA was used for statistical analyses. *P* < 0.05 was considered statistically significant. Statistical analyses were performed using SPSS 17.0 (SPSS Inc., Chicago, IL).

## 3. Results

### 3.1. Establishment of the GFP-Positive Chimeric Model

**Figure 1:**
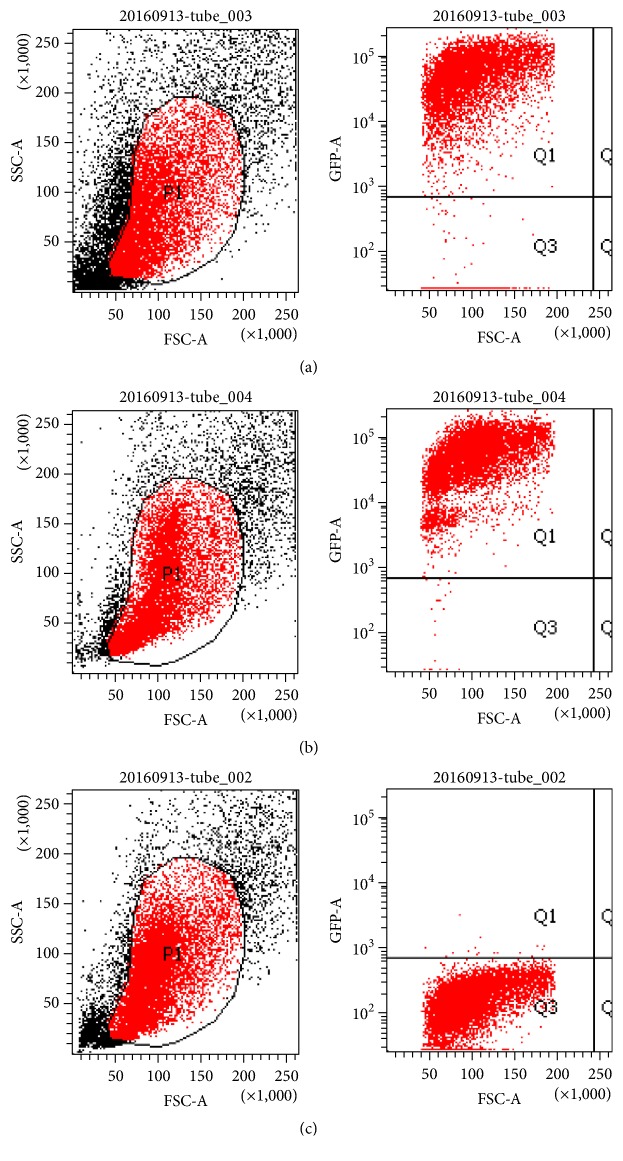
The percentage of GFP^+^ cells in a chimeric mouse (a), a GFP-transgenic mouse (b), and a wild-type C57BL/6 mouse (c) determined by flow cytometry. The following gating strategy was applied to determine the proportion of GFP^+^ cells among mononuclear cells: P1, active cells and Q1, GFP^+^cells. SSC, side scatter; FSC, forward scatter. The percentage of GFP^+^ cells was 90.8% in the chimeric mouse, 99% in the positive control GFP-transgenic mouse, and 0.1% in the negative control wild-type C57BL/6 mouse. The bone marrow cells of the recipient mice were almost completely replaced by GFP^+^ BMDCs from the donor mice.

We established a bone marrow transplantation chimeric mouse model with GFP-transgenic mice. A month after bone marrow transplantation, we examined the extent of chimerism in the mice. The average proportion of GFP^+^ cells was approximately 90.0% in the chimeric mice. Representative images are shown in Figure 1. The proportions of GFP^+^ cells among bone marrow mononuclear cells were approximately 90.8%, 99.0%, and 0.1% in chimeric, GFP-transgenic, and wild-type C57BL/6 mice, respectively. Recipient bone marrow cells were almost replaced by donor GFP^+^ BMDCs. Thus, the GFP^+^ chimeric mouse model was successfully established.

### 3.2. Blood Glucose

**Figure 2:**
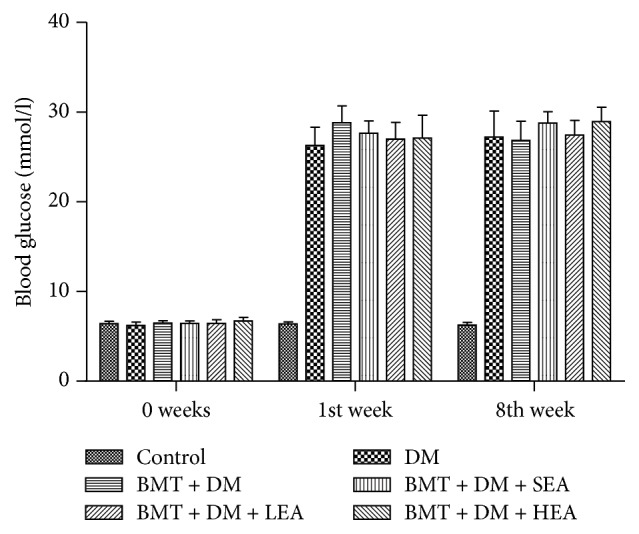
Blood glucose levels in the different treatment groups. All diabetic mice displayed significantly increased blood glucose levels. No differences in blood glucose levels were found between the DM group and the BMT + DM group at 0, 1, and 8 weeks. The blood glucose levels in the BMT + DM + SEA, BMT + DM + LEA, and BMT + DM + HEA groups did not change compared with those of the BMT + DM group at 0, 1, and 8 weeks.

As shown in Figure 2, the diabetic model was successfully established with high blood glucose levels in the DM group compared with those in the control group at 1 and 8 weeks (all *P* < 0.05). However, no differences in blood glucose levels were found between the DM group and the BMT + DM group at 0, 1, and 8 weeks (all *P* > 0.05). Blood glucose levels in the BMT + DM + SEA, BMT + DM + LEA, and BMT + DM + HEA groups did not change compared with those in the BMT + DM group at 0, 1, and 8 weeks (all *P* > 0.05).

### 3.3. Gastric Emptying

**Figure 3:**
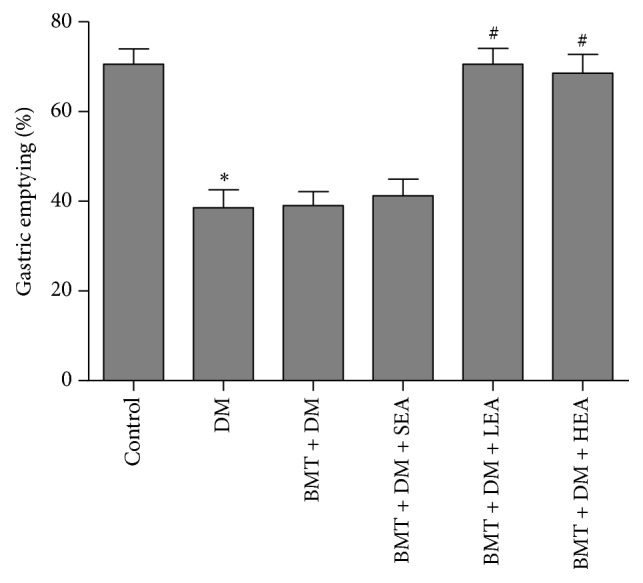
Effects of EA at ST36 on gastric emptying. Gastric emptying was significantly delayed in the DM group compared with that in the control group. No differences in gastric emptying were found between the DM group and the BMT + DM group. There was no significant difference between the BMT + DM group and the BMT + DM + SEA group. However, gastric emptying in the BMT + DM + LEA and BMT + DM + HEA groups was significantly accelerated compared with that in the BMT + DM group. ^*∗*^*P* < 0.05, compared with the control group. ^#^*P* < 0.05, compared with the BMT + DM group.

Figure 3 shows gastric emptying in different groups. Gastric emptying of the DM group was significantly delayed compared with that in the control group (*P* = 0.005). No differences in gastric emptying were found between the DM group and the BMT + DM group (*P* > 0.05). There was no significant difference between the BMT + DM group and the BMT + DM + SEA group (*P* > 0.05). However, gastric emptying of the BMT + DM + LEA and BMT + DM + HEA groups was significantly accelerated compared with that in the BMT + DM group (*P* = 0.002, *P* = 0.009).

### 3.4. Effects of EA on Bone Marrow-Derived ICC-MP and ICC-IM in the Chimeric Mouse Stomach

**Figure 4:**
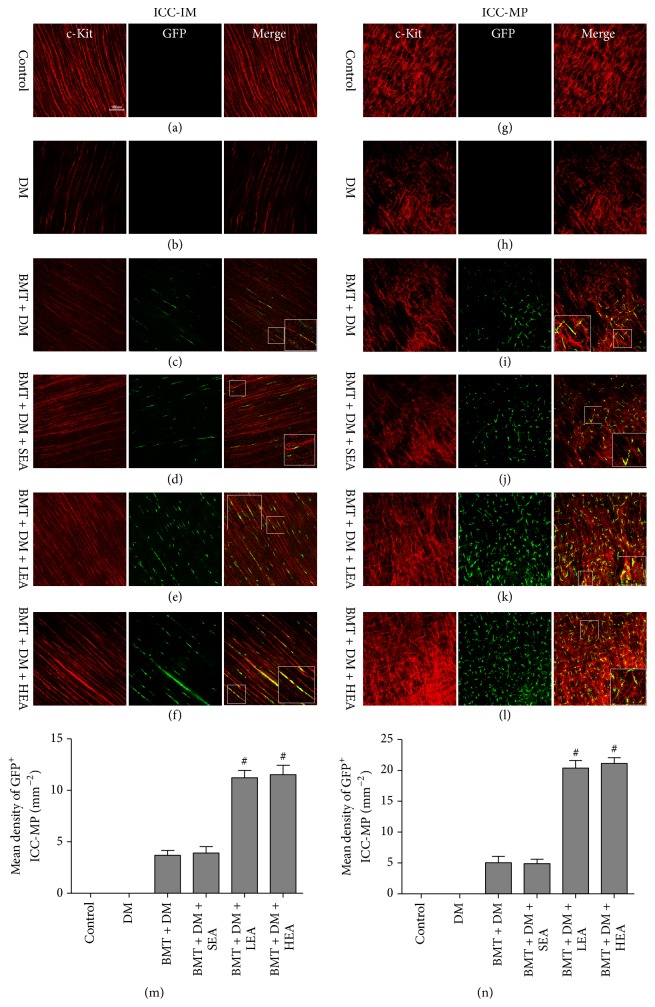
Immunofluorescence images of ICC-IM (a–f) and ICC-MP (g–l) in stomach corpus tissues labeled with antibodies against c-Kit (red) and GFP (green). c-Kit^+^GFP^+^ cells among ICC-IM and ICC-MP were not observed in the control group or the DM group. The number of c-Kit^+^GFP^+^ cells among ICC-IM and ICC-MP was markedly elevated in the BMT + DM + LEA and BMT + DM + HEA groups (*n* = 6 for each group) compared with that in the BMT + DM group. Quantitative analysis of GFP^+^ICC-IM and GFP^+^ICC-MP was performed in different groups in (m) and (n). ^#^*P* < 0.05, compared with the BMT + DM group. Scale bars = 100 *μ*m for all panels.

To assess the effects of EA on bone marrow-derived myenteric ICC (ICC-MP) and intramuscular (ICC-IM) in the stomach, we performed double-labeling of cells for c-Kit and GFP to identify bone marrow-derived ICC (Figure 4). c-Kit^+^GFP^+^ cells were not found among ICC-IM and ICC-MP in the control group or the DM group. However, c-Kit^+^GFP^+^ cells were observed in the muscle layer in the four chimeric groups. In response to electrical stimulation, numbers of c-Kit^+^GFP^+^ cells among ICC-IM and ICC-MP were markedly elevated in the BMT + DM + LEA and BMT + DM + HEA groups compared with those in the BMT + DM and BMT + DM + SEA groups (all *P* < 0.05). However, there was no significant difference between the BMT + DM group and the BMT + DM + SEA group (all *P* > 0.05).

**Figure 5:**
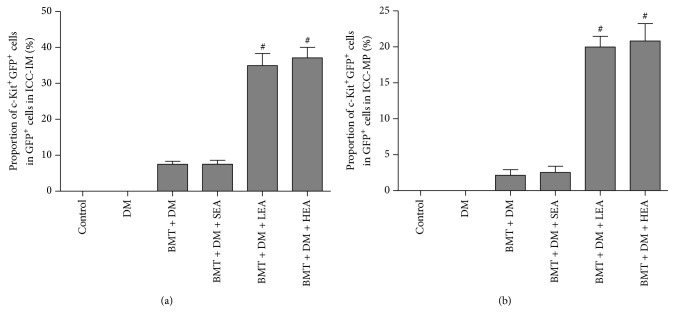
The proportion of bone marrow-derived cells differentiate into ICC networks in the corpus. c-Kit^+^GFP^+^ cells as bone marrow-derived ICC. GFP^+^ cells as bone marrow-derived cells. The proportions of c-Kit^+^GFP^+^ cells in GFP^+^ cells for ICC-IM and ICC-MP are increased in the BMT + DM + LEA and BMT + DM + HEA groups compared with that in the BMT + DM group. No differences were found between the BMT + DM group and the BMT + DM + SEA group (*n* = 6 for each group). ^#^*P* < 0.05, compared with the BMT + DM group.

To determine the contribution of BMDCs to ICC in the stomach, c-Kit^+^GFP^+^ cells were considered bone marrow-derived ICC and GFP^+^ cells were considered BMDCs, and we quantified the proportion of c-Kit^+^GFP^+^ cells in the GFP^+^ cells. As shown in Figure 5, the proportions of c-Kit^+^GFP^+^ cells in the GFP^+^ cells were increased in the BMT + DM + LEA and BMT + DM + HEA groups compared with those in the BMT + DM group for ICC-IM and ICC-MP (all *P* < 0.05). No differences were found between the BMT + DM group and the BMT + DM + SEA group for ICC-IM and ICC-MP (both *P* > 0.05).

**Figure 6:**
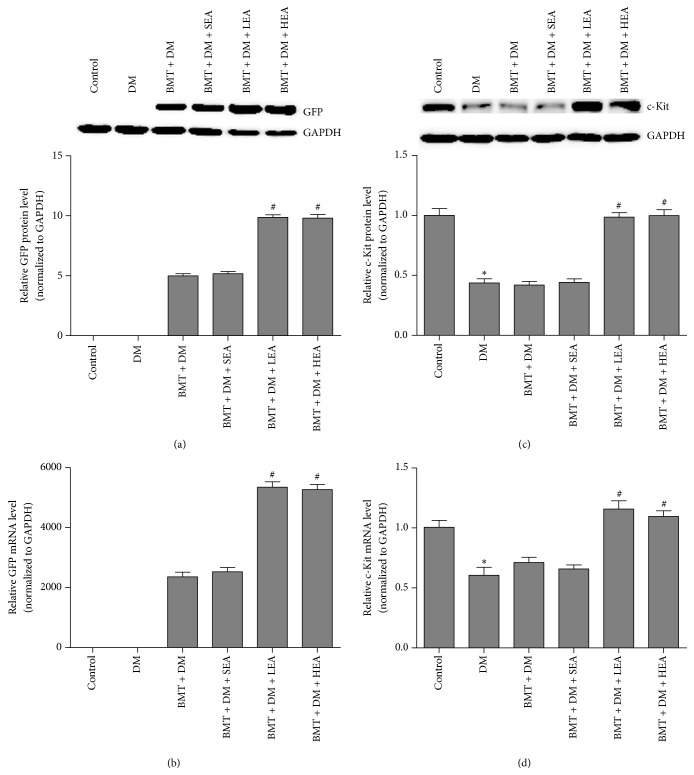
Protein and mRNA expression of GFP (a, b) and c-Kit (c, d) in stomach tissue. GFP was not expressed in the control group or the DM group. Compared with the BMT + DM group, the protein and mRNA levels of GFP were significantly increased in the BMT + DM + LEA and BMT + DM + HEA groups. In addition, compared with those in the control group, the protein and mRNA levels of c-kit were significantly decreased in the DM group. Significant increases in c-Kit protein and mRNA levels were also observed in the BMT + DM + LEA and BMT + DM+ HEA groups (*n* = 5 for each group) compared with those in the BMT + DM group. ^*∗*^*P* < 0.05, compared with the control group. ^#^*P* < 0.05, compared with the BMT + DM group.

The expression of GFP and c-Kit in the stomach was detected at both the protein and mRNA levels (Figure 6). GFP was not expressed in the control group or the DM group at the protein level. GFP protein levels were significantly increased in the BMT + DM + LEA and BMT + DM + HEA groups compared with those in the BMT + DM group (both *P* < 0.05). However, there was no significant difference in GFP protein levels between the BMT + DM group and the BMT + DM + SEA group (*P* = 0.983). On the other hand, c-Kit protein levels were significantly lower in the DM group than those in the control group (*P* = 0.001). c-Kit protein levels were significantly increased in the BMT + DM + LEA (*P* < 0.05) and BMT + DM + HEA (*P* < 0.05) groups compared with those in the BMT + DM group. However, no differences in c-Kit protein levels were found between the BMT + DM group and the BMT + DM + SEA group (*P* > 0.05). Similar results were observed for GFP and c-Kit mRNA levels.

### 3.5. Effects of EA on the SDF/CXCR4 Pathway

**Figure 7:**
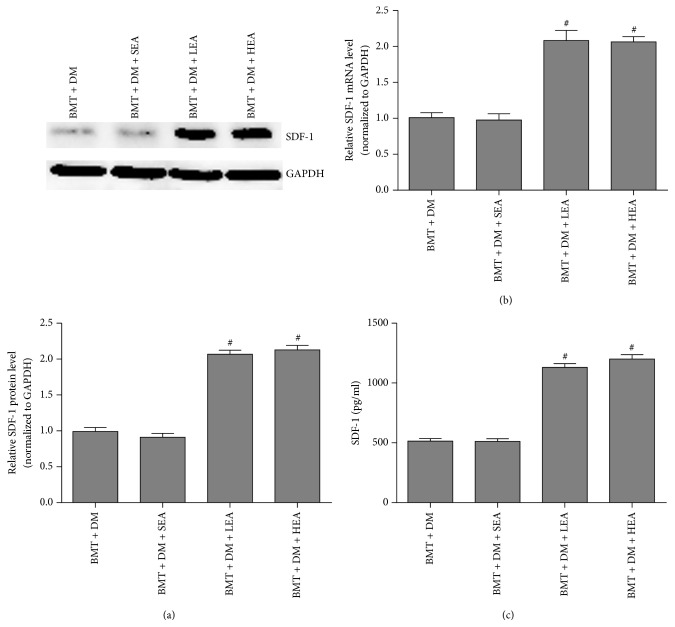
SDF-1 protein and mRNA levels in stomach tissue (a, b) and SDF-1 protein levels in the serum (c). SDF-1 protein and mRNA expression levels in stomach tissue were distinctly increased in the BMT + DM + LEA and BMT + DM + HEA groups compared with those in the BMT + DM group (*n* = 5 for each group). SDF-1 protein levels in the sera were distinctly increased in the BMT + DM + LEA and BMT + DM + HEA groups (*n* = 6 for each group) compared with those in the BMT + DM group. ^#^*P* < 0.05, compared with the BMT + DM group.

Figure 7 shows SDF-1 protein and mRNA levels in the stomach and SDF-1 protein levels in mouse sera. SDF-1 protein levels determined by western blotting were distinctly increased in the BMT + DM + LEA (*P* < 0.05) and BMT + DM + HEA (*P* < 0.05) groups compared with those in the BMT + DM group. No difference in SDF-1 protein levels was observed between the BMT + DM group and the BMT + DM + SEA group (*P* = 0.925). Similar results were also observed for SDF-1 mRNA levels determined by qRT-PCR in stomach tissue. In addition, SDF-1 protein levels in the serum detected by ELISA were 514.33 ± 20.86 pg/ml, 512.33 ± 21.52 pg/ml, 1131.17 ± 30.20 pg/ml, and 1200.67 ± 35.72 pg/ml in the BMT + DM, BMT + DM + SEA, BMT + DM + LEA, and BMT + DM + HEA groups, respectively. SDF-1 protein levels in the sera were markedly increased in the BMT + DM + LEA (*P* < 0.05) and BMT + DM + HEA (*P* < 0.05) groups compared with those in the BMT + DM group.

**Figure 8:**
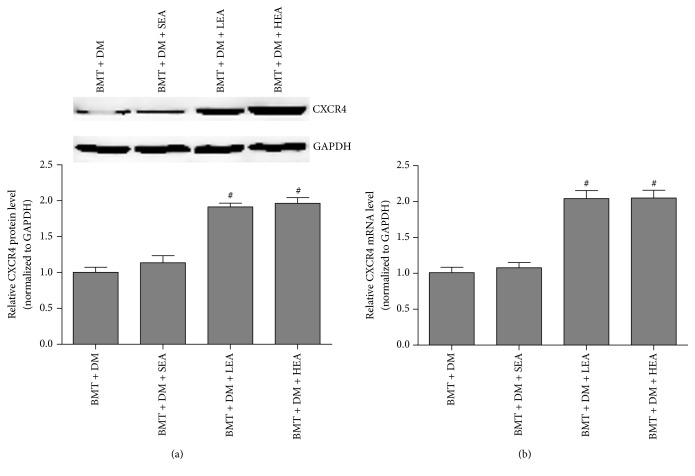
CXCR4 protein and mRNA expression in stomach tissue (a, b). CXCR4 protein and mRNA levels in stomach tissues were significantly increased in the BMT + DM + LEA and BMT + DM + HEA groups compared with those in the BMT + DM group (*n* = 5 for each group). ^#^*P* < 0.05, compared with the BMT + DM group.

Furthermore, CXCR4 protein and mRNA expression were detected by western blotting and qRT-PCR, respectively (Figure 8). CXCR4 protein levels in the stomach tissues were significantly increased in the BMT + DM + LEA (*P* < 0.05) and BMT + DM + HEA (*P* < 0.05) groups compared with those in the BMT + DM group. No difference was observed between the BMT + DM group and the BMT + DM + SEA group (*P* = 0.889). Similar changes were also observed for CXCR4 mRNA levels.

### 3.6. Effects of EA on the mSCF/Kit-ETV1 Signaling Pathway

**Figure 9:**
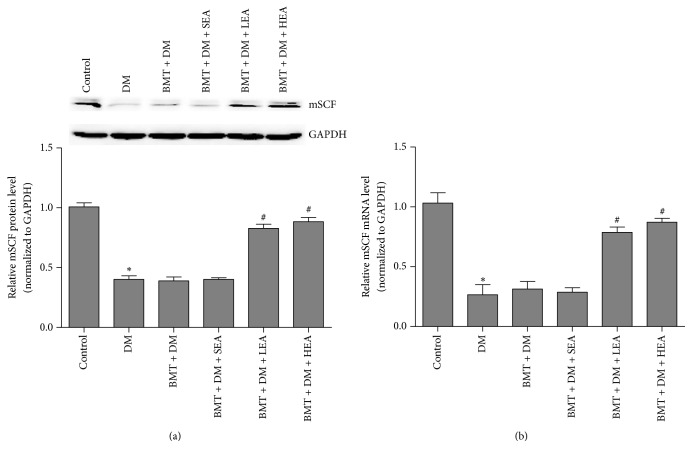
mSCF protein and mRNA expression (a, b) in stomach tissue. A distinct downregulation of mSCF protein and mRNA levels was observed in the DM group compared with that in the control group. Conversely, mSCF protein and mRNA levels were markedly increased in the BMT + DM + LEA and BMT + DM + HEA groups compared with those in the BMT + DM group (*n* = 5 for each group). ^*∗*^*P* < 0.05, compared with the control group. ^#^*P* < 0.05, compared with the BMT + DM group.

**Figure 10:**
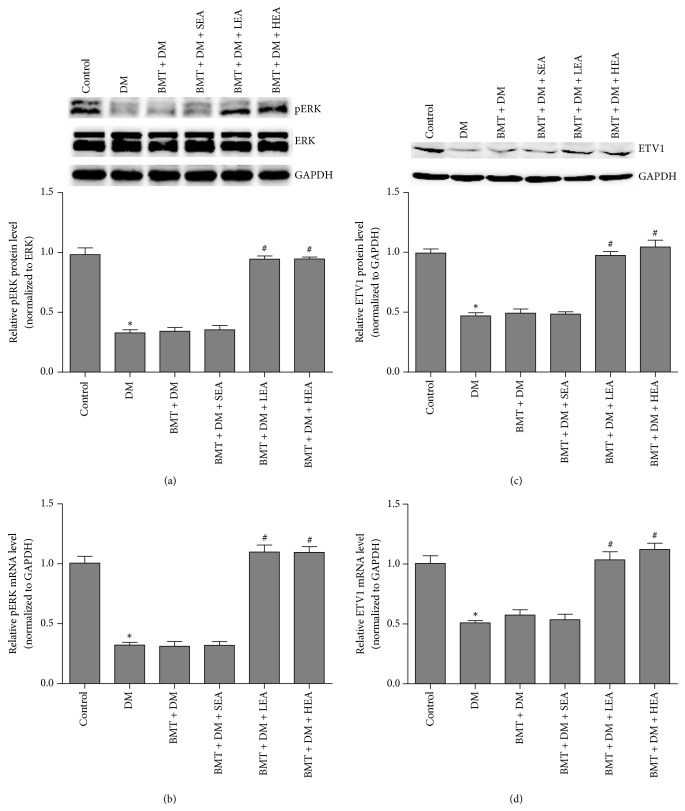
pERK (a, b) and ETV1 (c, d) protein and mRNA expression in stomach tissue. A distinct downregulation in pERK and ETV1 protein and mRNA levels was observed in the DM group compared with that in the control group. Conversely, pERK and ETV1 protein and mRNA levels were markedly increased in the BMT + DM + LEA and BMT + DM + HEA groups compared with those in the BMT + DM group (*n* = 5 for each group). ^*∗*^*P* < 0.05, compared with the control group. ^#^*P* < 0.05, compared with the BMT + DM group.

Figures 9 and 10 show mSCF, pERK, and ETV1 expression levels. A distinct downregulation of mSCF protein and mRNA levels was observed in the DM group compared with that in the control group (both *P* < 0.05). Conversely, SCF protein and mRNA levels were markedly increased in the BMT + DM + LEA (both *P* < 0.05) and BMT + DM + HEA (both *P* < 0.05) groups compared with those in the BMT + DM group. However, no significant differences were found between the BMT + DM and BMT + DM + SEA groups (both *P* > 0.05).

A distinct downregulation of pERK protein and mRNA levels was observed in the DM group compared with those in the control group (both *P* < 0.05). Conversely, pERK protein and mRNA levels were markedly increased in the BMT + DM + LEA (both *P* < 0.05) and BMT + DM + HEA (both *P* < 0.05) groups compared with those in the BMT + DM group. However, no significant differences were found between the BMT + DM and BMT + DM + SEA groups (both *P* > 0.05).

ETV1 protein and mRNA levels were also measured by western blotting and qRT-PCR, respectively. ETV1 protein expression in the DM group was markedly lower than that the control group (*P* < 0.05). ETV1 protein levels were markedly elevated in the BMT + DM + LEA and BMT + DM + HEA groups compared with those in the BMT + DM group (both *P* < 0.05). However, no significant differences were found between the BMT + DM and BMT + DM + SEA groups (*P* > 0.05). ETV1 mRNA levels exhibited a similar trend as that observed for ETV1 protein levels in the stomach.

## 4. Discussion

In the current study, both LEA and HEA at ST36 increased the number of bone marrow-derived ICC in the stomachs of diabetic mice, which may result in improved gastric emptying. The effects of EA were partly associated with enhanced SDF-1/CXCR4 and mSCF/Kit-ETV1 signaling pathways.

Delayed gastric emptying was improved, and c-Kit expression was increased after EA stimulation. In traditional Chinese medicine, acupuncture at ST36 is performed to treat gastrointestinal illness. EA is an advanced form of traditional acupuncture. In our previous study, both LEA (10 Hz) and HEA (100 Hz) at ST36 were found to improve gastroparesis in diabetic rats [4]. Therefore, we continued to use these two frequencies for EA stimulation in our current study. ICC is known to play an important role in the pathogenesis of diabetic gastroparesis. In our previous study, EA at ST36 ameliorated gastric emptying and sustained ICC networks in the diabetic rat gut [4]. In this study, EA at ST36 also improved delayed gastric emptying and increased the expression of c-Kit, suggesting that the effects of EA on gastroparesis may be mediated through ICC, resulting in improved gastric emptying.

Blood glucose levels did not change significantly after bone marrow transplantation and after EA stimulation. Hess et al. [20] showed that pancreatic engrafting cells derived from donor bone marrow cells reversed hypoinsulinemia and hyperglycemia caused by pancreatic damage. This conclusion is inconsistent with our findings. The reason for the inconsistency may be that the protocols were different. We induced our diabetic models via a single injection of high-dose STZ, and Hess et al. induced their diabetic models via multiple injections of low-dose STZ. On the other hand, we performed bone marrow transplant on mice before inducing our diabetic models and Hess et al. first induced diabetic models before bone marrow transplantation. This difference indicates that EA does not sustain ICC networks by lowering blood glucose but directly through the ICC.

EA increased the number of ICC-IM and ICC-MP derived from bone marrow cells. Replenishment from stem cells and ICC proliferation increase the number of ICC. In recent years, some studies have shown that BMDCs differentiate into ICC in the intestinal tract. Ishii et al. [21] showed that BMDCs incorporate into ICC networks and improve dysmotility in W/W(v) mice. A study conducted by Su et al. [7] suggested that decreased homing of cells from the bone marrow may be a major cause of ICC loss in the intestines of mice with diabetes mellitus. Another recent study using GFP-labeled BMDCs transplantation successfully identified BMDCs as the origin of ICC in the small intestine [8]. However, whether BMDCs differentiate into gastric ICC has not been reported. This is the first study demonstrating the presence of GFP^+^c-Kit^+^ cells in the stomach using chimeric diabetic mice. Studies have reported that EA efficiently promotes the differentiation of bone marrow mesenchymal stem cells into neuron-like cells and chondrocytes [9, 22]. However, whether EA promotes the differentiation of BMDCs into ICC is not yet known. In this study, the number of ICC-IM and ICC-MP derived from bone marrow cells increased after EA stimulation, indicating that both HEA and LEA increase bone marrow-derived ICC-IM and ICC-MP numbers in the stomach of diabetic mice.

SDF-1 and CXCR4 expression levels in chimeric diabetic mice were significantly increased after EA stimulation. Chemokines are essential for the migration of bone marrow stem cells to the stomach. The chemokine SDF-1 and its ligand CXCR4 are crucial for the homing of hematopoietic stem/progenitor cells [23]. Kaku et al. [24] observed the recruitment of BMDCs to periodontal ligaments via the SDF-1/CXCR4 axis. A recent report showed that blocking SDF-1/CXCR4 inhibited bone marrow-derived pericyte differentiation [25]. However, it is unknown whether the SDF-1/CXCR4 signaling pathway mediates the EA-induced promotion of BMDCs migration to the stomach. In our study, SDF-1 and CXCR4 expression levels in chimeric diabetic mice were significantly increased after EA stimulation at various frequencies, indicating that both HEA and LEA promote the migration of bone marrow cells to the stomach through the SDF-1/CXCR4 signaling pathway.

The expression of mSCF and c-Kit was increased after EA stimulation at various frequencies. As the ligand of c-Kit, SCF also plays an important role in the survival and proliferation of ICC. The development and maintenance of ICC have been shown to be dependent on SCF, particularly mSCF via c-Kit [26, 27]. In our previous study, EA increased mSCF levels in diabetic rats [18]. However, the expression of c-kit and mSCF in chimeric mice has not yet been studied. In the current study, both HEA and LEA increased mSCF and c-Kit expression, indicating that EA may promote the maintenance of bone marrow-derived ICC via mSCF/c-Kit signaling.

HEA and LEA increased pERK and ETV1 expression in chimeric diabetic mice. ETV1 belongs to the ETS family of proteins. Inhibition of Kit signaling results in the rapid loss of ETV1 [28], indicating that ETV1 is a major downstream effector of mSCF/Kit signaling. ETV1 is highly expressed in various subtypes of ICC, and a significant loss of ICC-IM and ICC-MP has been observed in ETV1-knockout mice, indicating that ETV1 may be selectively required for the development of ICC-MP and ICC-IM [14]. However, the ETV1 protein is very unstable and maintained by Kit-ERK signaling [29]. In our previous study, ETV1 expression in diabetic mice was markedly increased after EA stimulation, and EA maintained ICC networks via the mSCF/Kit-ETV1 pathway [15]. In the current study, both HEA and LEA elevated the expression of c-Kit, mSCF, pERK, and ETV1 in chimeric diabetic mice, suggesting that the mSCF/Kit-ETV1 signaling pathway likely mediates the effects of EA stimulation on the maintenance of bone marrow-derived ICC.

## 5. Conclusion

In conclusion, both LEA and HEA at ST36 increase the number of bone marrow-derived ICC in the stomachs of diabetic mice at least in part through the SDF-1/CXCR4 and mSCF/c-Kit-ETV1 signaling pathways, thereby maintaining ICC networks and ameliorating gastric emptying. Therefore, EA may exert promising therapeutic effects on gastroparesis. These findings provide new insights into the therapeutic effects of EA on gastrointestinal motility diseases. However, the mechanisms by which EA increases the number of bone marrow-derived ICC must be investigated in the future.
